# Wood dust and risk of leukemia: Systematic review and meta-analysis

**DOI:** 10.1371/journal.pone.0307444

**Published:** 2024-08-27

**Authors:** Yaser Soleimani, Mahdi Daraei, Parniyan Sadeghi, Alireza Khazali, Hanieh Rostami, Sheyda Mahmoudi, Alireza Mosavi Jarrahi, Mohammad Reza Taherian, Goljamal Jorjani, Nasser Bahari

**Affiliations:** 1 Medical School, Shahid Beheshti University of Medical Sciences, Tehran, Iran; 2 Cancer Research Center, Shahid Beheshti University of Medical Sciences, Tehran, Iran; 3 Department of Epidemiology, School of Public Health and Safety, Shahid Beheshti University of Medical Sciences, Tehran, Iran; Bindura University of Science Education, SOUTH AFRICA

## Abstract

**Objectives:**

This study aimed to perform a systematic review and meta-analysis to investigate the relationship between wood dust exposure and leukemia. The objectives included synthesizing available evidence, assessing its quality, identifying potential sources of heterogeneity, and drawing conclusions regarding the association between wood dust and leukemia.

**Methods:**

A systematic literature search was conducted to identify studies meeting that report on the association between wood dust and leukemia. The Joanna Briggs Institute Critical Appraisal tools were employed to ensure robust quality assessment. Meta-analysis, using random-effects models, synthesized evidence from studies with low risk of bias. Overall odds ratios (ORs) and 95% confidence intervals (CIs) were calculated. Subgroup analyses explored potential sources of heterogeneity.

**Results:**

The meta-analysis included a comprehensive review of various study types, encompassing 7 studies that examined the association between wood dust exposure and leukemia risk. The analysis revealed a statistically significant positive association, with an overall odds ratio (OR) of 1.56 (95% CI: 1.15–2.12). This indicates that individuals exposed to wood dust are 1.56 times more likely to develop leukemia compared to those not exposed, with the 95% confidence interval ranging from 1.15 to 2.12, highlighting a substantial risk elevation across different study designs. Quality assessment using The Joanna Briggs Institute Critical Appraisal tools demonstrated a low risk of bias across all included studies, enhancing the credibility of the observed association. Subgroup analyses were conducted to explore potential sources of heterogeneity within the studies. Notably, subgroup analysis based on the year of the study revealed significant differences, as indicated by an I^2 value of 87%. The robustness of these results underscores the importance of addressing wood dust exposure as an occupational hazard, particularly in industries related to woodworking and forestry.

**Conclusion:**

This meta-analysis provides robust evidence supporting an increased risk of leukemia associated with wood dust exposure implying proactive measures in people exposed to dust.

## Introduction

Leukemia, characterized by the abnormal proliferation of white blood cells in the bone marrow and bloodstream, represents a significant public health challenge worldwide. Over the decade spanning from 2006 to 2016, there has been a noticeable surge in leukemia cases among children [1] and adult, positioning it as the 15th most prevalent cancer globally [2]. Additionally, it stands as the 11th leading cause of cancer-related mortality. The intricate interplay of various factors contributes to the onset of leukemia, encompassing medical treatments, genetic predispositions [3], chemical exposures [4], and lifestyle choices [5].

Wood, a fundamental material with a diverse structural composition, is broadly classified into hardwoods and softwoods. During the processing of hardwoods, particulate matter in the form of dust is generated, a byproduct that has garnered recognition as carcinogenic by the International Agency for Research on Cancer (IARC). The implications of wood dust exposure on worker health are considerable, with prevalence rates of associated health issues ranging from 10% to 15%. While certain cancers, notably nasal cavity and lung cancer, exhibit clear associations with wood dust exposure, the link to leukemia remains less unequivocal. Existing research predominantly hinges on epidemiological studies to elucidate the potential influence of wood dust exposure on leukemia development [6–8]. However, the outcomes of these studies frequently yield conflicting results, fostering ongoing debates regarding the precise nature of the relationship between wood dust exposure and leukemia incidence.

The uncertainty of the relationship between wood dust exposure and leukemia highlights the need for a better understanding of this relationship. This understanding has important implications in the development of preventive measures, occupational safety procedures and early detection strategies [9]. In response to these imperatives, a systematic review and meta-analysis were conducted to ascertain whether exposure to wood dust heightens the risk of leukemia across diverse demographic strata, encompassing age, gender, race, and ethnicity.

The general objectives of this study include conducting a comprehensive review of existing data on the relationship between wood dust and leukemia, combining currently available data for the collection of scientific data, investigating the carcinogenic role of wood dust in the pathogenesis of leukemia, and investigating the carcinogenic role of wood dust in the pathogenesis of leukemia. By examining the complexities of wood dust exposure and its potential link to leukemia, this study seeks to provide valuable information for public health strategies, testing and improving occupational safety.

This systematic review and evaluation represent a significant effort to reveal the relationship between wood dust and leukemia and provides a platform for evidence-based decision-making in workplace and public health measures. By collecting existing evidence and resolving uncertainties, this study aims to provide better protection in action work and improve safety standards, thus protecting the health of workers and the general public against the dangers of wood dust.

## Materials and methods

### Study design and selection criteria

A comprehensive detail of the protocol of this study has been already published [10]. The study aimed to comprehensively evaluate the association between wood dust exposure and the risk of leukemia through a systematic review and meta-analysis. The selection criteria included:

• Publication Source: Articles from peer-reviewed journals in English were included to ensure scientific rigor and accessibility. Although we acknowledge that excluding non-English articles can introduce publication bias, our search did not identify any relevant articles in other languages that met our criteria.

• Study Design: Observational studies (cohort, case-control, cross-sectional) were considered to provide diverse yet focused analysis. These study designs were included because they are well-suited for identifying associations and patterns in real-world settings, which is crucial for understanding the epidemiology and risk factors of leukemia. Meta-analyses and systematic reviews were excluded to maintain focus on individual research studies.

• Population: Studies involving human subjects exposed to wood dust in occupational settings were included due to their relevance.

• Outcome of Interest: Emphasis was placed on leukemia, including subtypes like Acute Lymphoblastic Leukemia (ALL), Acute Myeloid Leukemia (AML), Chronic Lymphocytic Leukemia (CLL), and Chronic Myeloid Leukemia (CML) to capture the disease’s breadth.

### Search strategy

A comprehensive literature search was conducted until 03/11/2023 in PubMed, Scopus, and Web of Science databases. Customized search terms for each database are as follows:

PubMed (1271 Articles):

("Wood Dust Exposure" OR "Occupational Hazards" OR "Woodworking Industry" OR "Occupational Exposure" OR "Wood Dust Particles" OR "Wood Dust Inhalation" OR "Woodworking Occupations" OR "Sawdust Exposure") AND ("Leukemia" OR "Hematological Malignancies" OR "Blood Cancer" OR "Myeloid Leukemia" OR "Lymphocytic Leukemia" OR "Leukemogenesis" OR "Leukemia Risk Factors" OR "Leukemia Pathogenesis" OR "Hematopoietic Cancer" OR "Blood Cell Cancer").

Scopus (41 Articles):

TITLE-ABS-KEY (wood AND dust) AND TITLE-ABS-KEY ("leukemia" OR "blood and cancer" OR "hematological and malignancies" OR "acute and lymphoblastic and leukemia" OR "acute and myeloid and leukemia" OR "chronic and lymphocytic and leukemia" OR "chronic and myeloid and leukemia").

Web of Science (544 Articles):

(TS = (Wood OR dust)) AND TS = (blood cancer OR hematological malignancies OR acute lymphoblastic leukemia OR acute myeloid leukemia OR chronic lymphocytic leukemia OR chronic myeloid leukemia OR leukemia).

### Information selection and extraction

In adherence to the Preferred Reporting Items for Systematic Reviews and Meta-Analyses (PRISMA) guidelines [11], a meticulous approach was undertaken to select and extract relevant information from identified studies.

Two independent reviewers meticulously screened the titles and abstracts of the initially identified articles for eligibility, ensuring alignment with the predetermined inclusion criteria. Any discrepancies or disagreements were resolved through discussion and consensus.

Following the initial screening phase, full-text articles of potentially relevant studies were retrieved and further evaluated against the inclusion criteria. The inclusion criteria encompassed parameters such as study design, population characteristics, exposure details, outcome measures, and language of publication. To enhance the comprehensiveness of our search, we conducted additional searches in specialized registers, consulted with experts in the field, and reviewed the reference lists of relevant articles. Despite these efforts, no additional studies meeting our inclusion criteria were identified. While we employed a comprehensive search strategy, including unpublished studies and gray literature, the possibility of unreported or unpublished studies with null findings impacting our overall conclusions cannot be entirely ruled out.

Data extraction was conducted independently by two reviewers using standardized forms to ensure consistency and accuracy. Extracted data encompassed various aspects, including study characteristics (e.g., author(s), publication year), participant demographics, details of wood dust exposure, leukemia outcomes, and effect size measures (e.g., Odds Ratios and 95% Confidence Intervals). Although we focused on English-language studies, no studies in other languages were identified that met our inclusion criteria.

Moreover, adherence to PRISMA guidelines facilitated transparency and comprehensiveness throughout the information selection and extraction process. By following these established guidelines, the study aimed to minimize bias and enhance the reliability of the synthesized evidence regarding the association between wood dust exposure and leukemia risk.

### Scientific evidence

Quality assessment was conducted using the Joanna Briggs Institute Critical Appraisal of Case-Control Studies tool. Meta-analysis employed Odds Ratios (OR) and corresponding 95% Confidence Intervals (CI) as effect size measures. A random-effects model synthesized ORs to generate an overall pooled estimate.

### Meta-analysis

The meta-analysis was conducted using both Meta-R and Stata applications to ensure robustness and reliability in synthesizing the collected evidence.

Meta-R Application [12]: In the Meta-R application, the pooled effect size estimate was calculated using a random-effects model, which appropriately accounted for heterogeneity across studies. The application facilitated the aggregation of individual study findings, generating an overall estimate of the association between wood dust exposure and leukemia risk. This approach provided a comprehensive summary of the collective evidence, enhancing the statistical power of the analysis.

### Stata application (version 17)

Similarly, in the Stata application, the meta-analysis was conducted using a random-effects model to combine effect sizes from individual studies. Stata’s intuitive interface allowed for seamless data manipulation and analysis, facilitating the synthesis of evidence from diverse sources. Additionally, sensitivity analyses were performed to assess the robustness of the findings, ensuring the reliability of the pooled effect size estimate.

### Integration of results

The results obtained from both Meta-R and Stata applications were integrated to provide a unified assessment of the association between wood dust exposure and leukemia risk. By leveraging the strengths of each application, including their advanced statistical capabilities and user-friendly interfaces, the meta-analysis produced comprehensive insights into the research question.

In addition to the primary meta-analysis, a regression-based Egger test was conducted to assess small-study effects. The test indicated evidence against the null hypothesis (H0: beta1 = 0), suggesting the presence of such effects. The coefficient (beta1) was -2.69 with a standard error (SE) of 1.091 and a Z-score of -2.46, yielding a statistically significant p-value of 0.0137. Heterogeneity was evaluated using the I^2 statistic, and meta-regression was performed to explore potential sources. Sensitivity analysis was conducted to assess the robustness of study results.

## Results

### Identification and selection of studies

**Fig 1:**
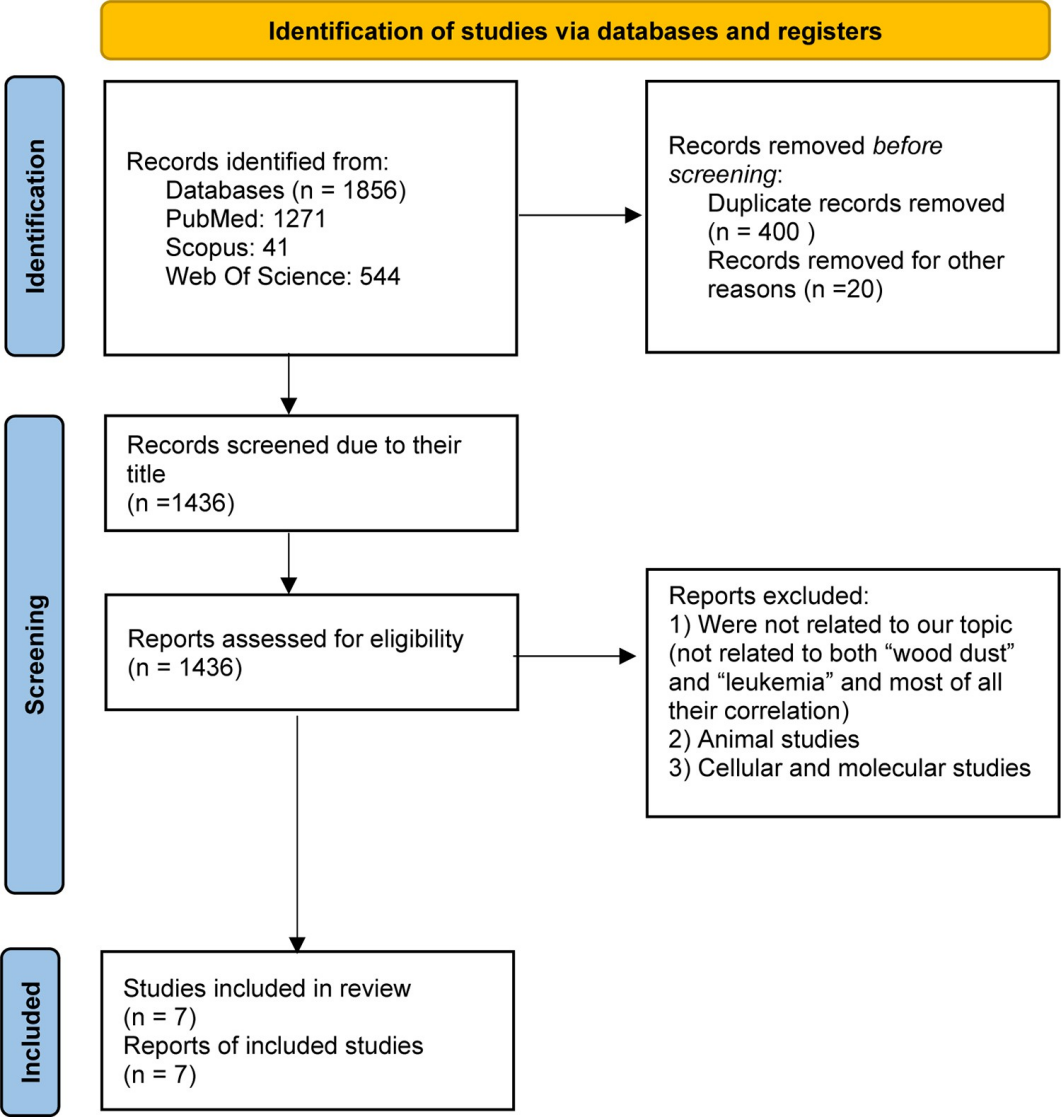
Flow diagram of systematic review and meta-analysis, wood dust and leukemia.

A comprehensive search across electronic databases, including PubMed, Scopus and Web Of Science, identified a total of 1856 potential studies related to wood dust exposure and leukemia risk. Following the removal of duplicates and a thorough screening process, 7 studies were deemed eligible for inclusion in the meta-analysis [13–18]. Reasons for study exclusion are detailed in the Preferred Reporting Items for Systematic Reviews and Meta-Analyses (PRISMA) flow diagram (Fig 1).

### Characteristics of included studies

(Table 1) summarizes the characteristics of the included studies, including author names, publication years, study designs, sample sizes, and key characteristics of the study populations.

**Table 1:** Summary of published results on the relationship between exposure to wood dust and risk of leukemia.

Reference	Quality Score	Country	Study Design	Source of funding	Age range	Leukemia Subtype	Exposure Assessment-Measurement Method	Diagnostic Criteria Used	Follow up Duration	Reported Results (Odds Ratio and 95% Confidence Intervals)
P A McKinney 1991 [13]	Low Risk	England	Case-control	The Leukaemia Research Fund	0–14 years	Childhood leukemia & *^1^ non-Hodgkin’s lymphoma	Face to Face Interview & Questionnaire & History of Working	Interview	15 years	OR: 1.49 95%CI:(0.8–2.76)
Timo Partanen 1993 [14]	Low Risk	Finland	Case-control	Grants from the Academy of Finland and the Finnish Work Environment Fund	Not mentioned	All subtypes of leukemia	Questionnaire	Hospital Data	40 years	OR:0.56 95%CI:(0.15–2.18)
Catherine Metayer(ALL) 2016 [15]	Low Risk	USA	Case-control	National Institute of Environmental Health Sciences, US	0–15 years	Acute Lymphoblastic Leukemia (ALL)	Interview	History & Clinical Diagnosis	8 years	OR: 1.41 95%CI:(0.92–2.16)
Catherine Metayer(AML) 2016 [15]	Low Risk	USA	Case-control	National Institute of Environmental Health Sciences, US	0–15 years	Acute Myeloid Leukemia (AML)	Interview	Clinical History & Clinical Diagnosis	8 years	OR: 1.77 95%CI:(0.83–3.78)
Julie Volk 2020 [16]	Low Risk	Denmark	Case-control	None	All	Chronic Lymphocytic Leukemia (CLL)	History of Working	Parents & Their Employment Histories	49 years	OR: 1.44 95%CI:(1.08–1.94)
Pierluigi Cocco 2021 [17]	Low Risk	Czech Republic, France, Germany, Ireland, Italy, Spain	Case-control	*^2^	All	All subtypes of leukemia	Questionnaire	Clinical History & Questionnaire	7 years	OR: 1.1 95%CI:(0.72–1.95)
Marios Rossides 2022 [18]	Low Risk	Sweden	Case-control	Swedish Research Council for Health, Working Life, and Welfare and the Swedish Research Council	0–19 years	All subtypes of leukemia	History of Working	History & Clinical Diagnosis	55 years	OR: 2.62 95%CI:(2.61–2.63)

*This study has reported combined data for leukemia and non-Hodgkin’s lymphoma.

*^2^ European Commission, 5th Framework Programme, Quality of Life (QLK4-CT-2000 00422); (ii) European Commission, 6th Framework Programme, FP6-2003-FOOD-2-B (contract No. 023103); (iii) Instituto de Salud Carlos III (Spanish Government) (PI17/01280 and CIBERESP) and by the Agència de Gestió d’Ajuts Universitaris i de Recerca (AGAUR), CERCA Programme / Generalitat de Catalunya for institutional support (grant 2017SGR1085); (iv) the German Federal Office for Radiation Protection (grants No. StSch4261 and StSch4420); (v) La Fondation de France; (vi) the Italian Ministry for Education, University and Research (PRIN 2007 prot. 2007WEJLZB and PRIN 2009 prot. 20092ZELR2); (vii) the Italian Association for Cancer Research (IG 2011/11855); (viii) Ministry of Health- Czech republic–Conceptual development of research organization (DRO) (Masaryk Memorial Cancer Institute MMCI, 00209805); and (ix) The Irish Health Research Board.

### Quality assessment

All the studies included for analysis were of the case-control design, allowing us to employ a consistent methodology for quality assessment across the entire set of studies.

The quality assessment of the case-control studies included in this systematic review was conducted using The Joanna Briggs Institute (JBI) Critical Appraisal tools specifically designed for Case Control Studies [19]. The rigorous evaluation aimed to assess the methodological robustness and potential biases within each study, contributing to the overall validity of our meta-analysis.

#### 1. Selection of cases and controls

The first criterion examined the clarity and appropriateness of the selection process for cases and controls. All included studies demonstrated a well-defined case definition and appropriate methods for selecting controls. The risk of bias related to the selection of cases and controls was deemed low across all studies. The number of cases and controls for each study is provided in Table 2.

**Table 2:** Patients with leukemia according to their occupational exposure from studies included in the meta-analysis.

Reference	Leukemia Subtypes Studied	Number of cases	Number of controls	Sample size (No. of cases + No. of controls)
* P A McKinney 1991 [13]	Childhood leukemia & * non-Hodgkin’s lymphoma	23	29	52
Timo Partanen 1993 [14]	All subtypes of leukemia	6	152	158
Catherine Metayer 2016 [15]	Acute Lymphoblastic Leukemia (ALL)	45	49	94
Catherine Metayer 2016 [15]	Acute Myeloid Leukemia (AML)	9	49	58
Julie Volk 2020 [16]	Chronic Lymphocytic Leukemia (CLL)	49	825	874
Pierluigi Cocco 2021 [17]	All subtypes of leukemia	58	276	334
Marios Rossides 2022 [18]	All subtypes of leukemia	346	7429	7775

*This study has reported combined data for leukemia and non-Hodgkin’s lymphoma.

#### 2. Comparability of cases and controls

The second criterion focused on the comparability of cases and controls in terms of potential confounding factors. All studies systematically addressed and controlled for confounding variables, such as age, gender, and relevant exposures. The comparability between cases and controls was consistently robust, resulting in a low risk of bias for this criterion.

#### 3. Exposure

The third criterion evaluated the measurement and ascertainment of wood dust exposure. All studies employed reliable methods to measure and ascertain exposure, including detailed exposure assessments and appropriate tools. The risk of bias related to exposure measurement was consistently low across all studies.

#### 4. Outcome

The fourth criterion scrutinized the reliability and validity of methods used to identify and confirm cases of leukemia. All studies exhibited a clear case definition, appropriate diagnostic criteria, and reliable outcome ascertainment. The risk of bias related to outcome ascertainment was uniformly low across the included studies.

#### 5. Statistical analysis

The final criterion assessed the appropriateness of statistical methods employed in analyzing the data. All studies used sound statistical methods, including appropriate tests, adjustments for potential confounders, and transparent reporting of results. The risk of bias related to statistical analysis was consistently low.

The overall assessment based on The Joanna Briggs Institute Critical Appraisal tools for Case Control Studies indicates that all included studies were at low risk of bias across all assessed criteria. The methodological quality of the case-control studies was consistently high, enhancing the reliability of the evidence synthesized in our meta-analysis.

The uniform low risk of bias across all assessed criteria highlights the methodological robustness of the included case-control studies. The studies consistently adhered to rigorous standards in selecting cases and controls, addressing comparability issues, measuring exposure, ascertaining outcomes, and conducting statistical analyses. This high-quality evidence contributes to the strength and reliability of the findings in our systematic review.

The low risk of bias among the included studies increases the reliability of our meta-analysis results. With high-quality evidence, the potential for systematic errors and biases influencing the overall findings is minimized. The robustness of the included studies enhances the confidence in the observed association between wood dust exposure and leukemia.

While the quality assessment results indicate a consistently low risk of bias across all studies, it is essential to acknowledge that no study is entirely devoid of limitations. The quality assessment tools rely on reported information, and the absence of reported biases does not guarantee the absence of all potential biases. However, the systematic use of JBI tools ensures a standardized and thorough evaluation of study quality.

### Overall meta-analysis

**Fig 2:**
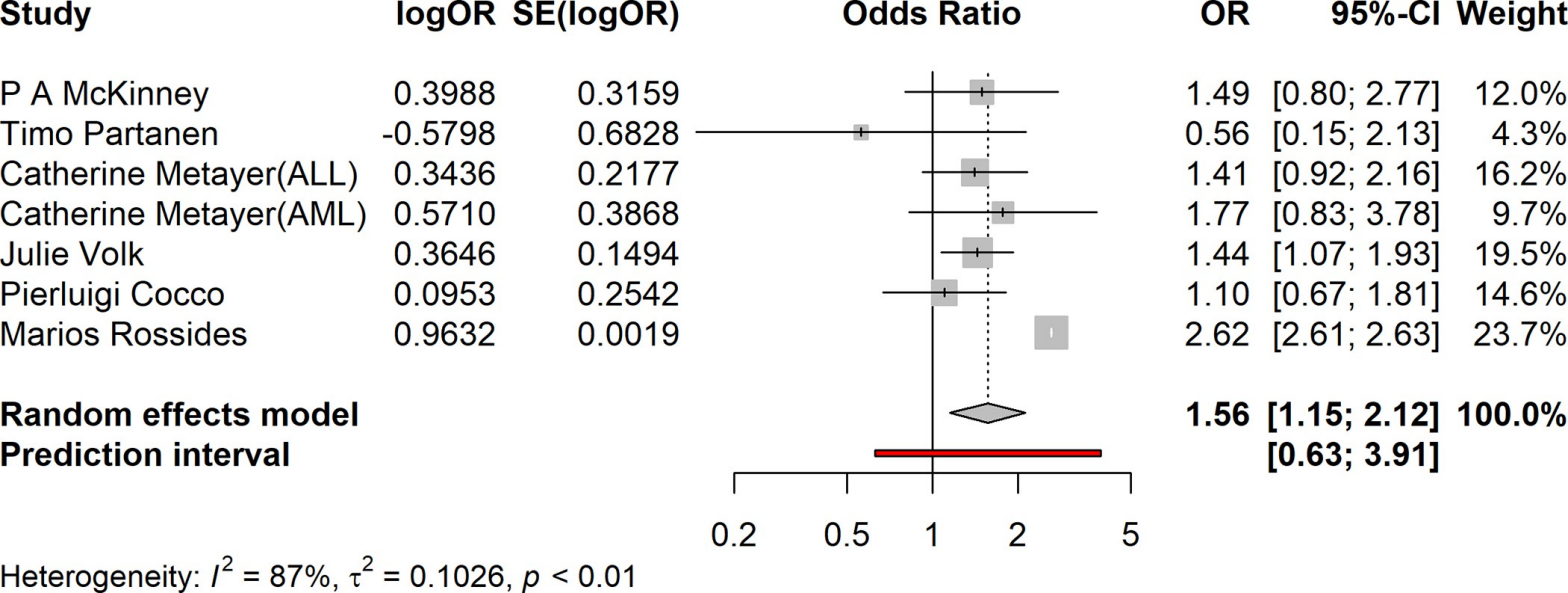
Forest plot, meta-analysis of wood dust exposure and risk of leukemia.

The pooled analysis of the included studies revealed OR: 1.56 (95% confidence interval [CI]: [1.15–1.12]) for the association between wood dust exposure and leukemia risk. The forest plot illustrating individual study effect sizes and the overall summary estimate is presented in Fig 2.

The Metayer study has presented findings for both types of Acute Lymphoblastic Leukemia (ALL) and Acute Myeloid Leukemia (AML), each with distinct Odds Ratios (ORs) and corresponding 95% Confidence Intervals (CIs). In order to enhance precision and minimize the likelihood of errors in data analysis, we have chosen to treat these results as separate entities.

### Subgroup analysis

In our meta-analysis, subgroup analysis was performed based on “publication year” to investigate changes in effect sizes over time. The test for group differences yielded a significant result, as evidenced by a test statistic (Q_b) of chi2(5) = 155.02, with a p-value (Prob > Q_b) of 0.000. This compelling finding indicates a noteworthy divergence in the observed effect across different publication years. The implications of this temporal variation underscore the dynamic nature of the phenomenon under investigation and emphasize the importance of considering the evolution of research findings over time. These results contribute valuable insights to our understanding of the trends and shifts in the studied variable, adding depth to the broader narrative within the field.

### Heterogeneity and sensitivity analysis

**Fig 3:**
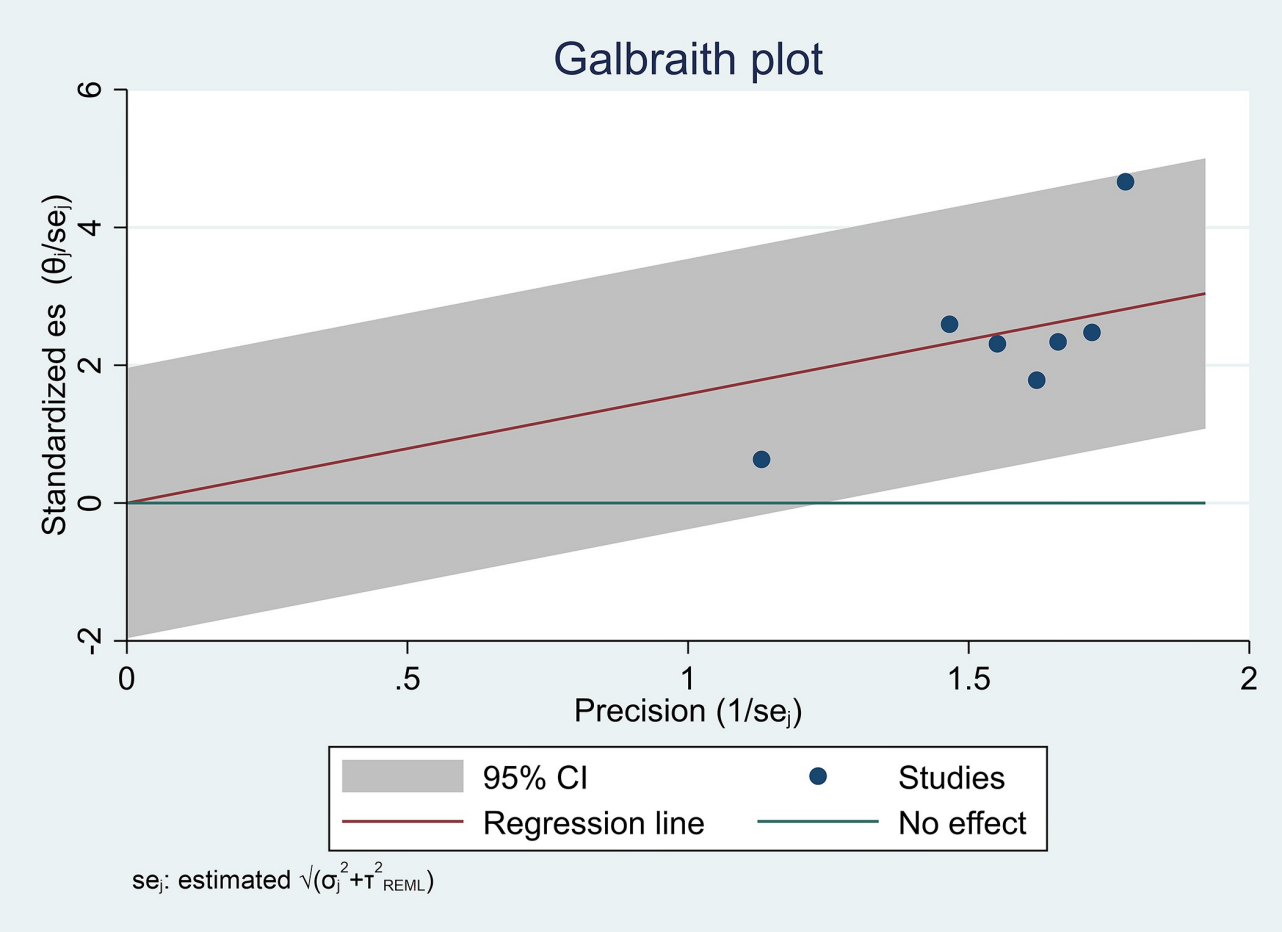
Galbraith plot, demonstrating heterogeneity.

**Fig 4:**
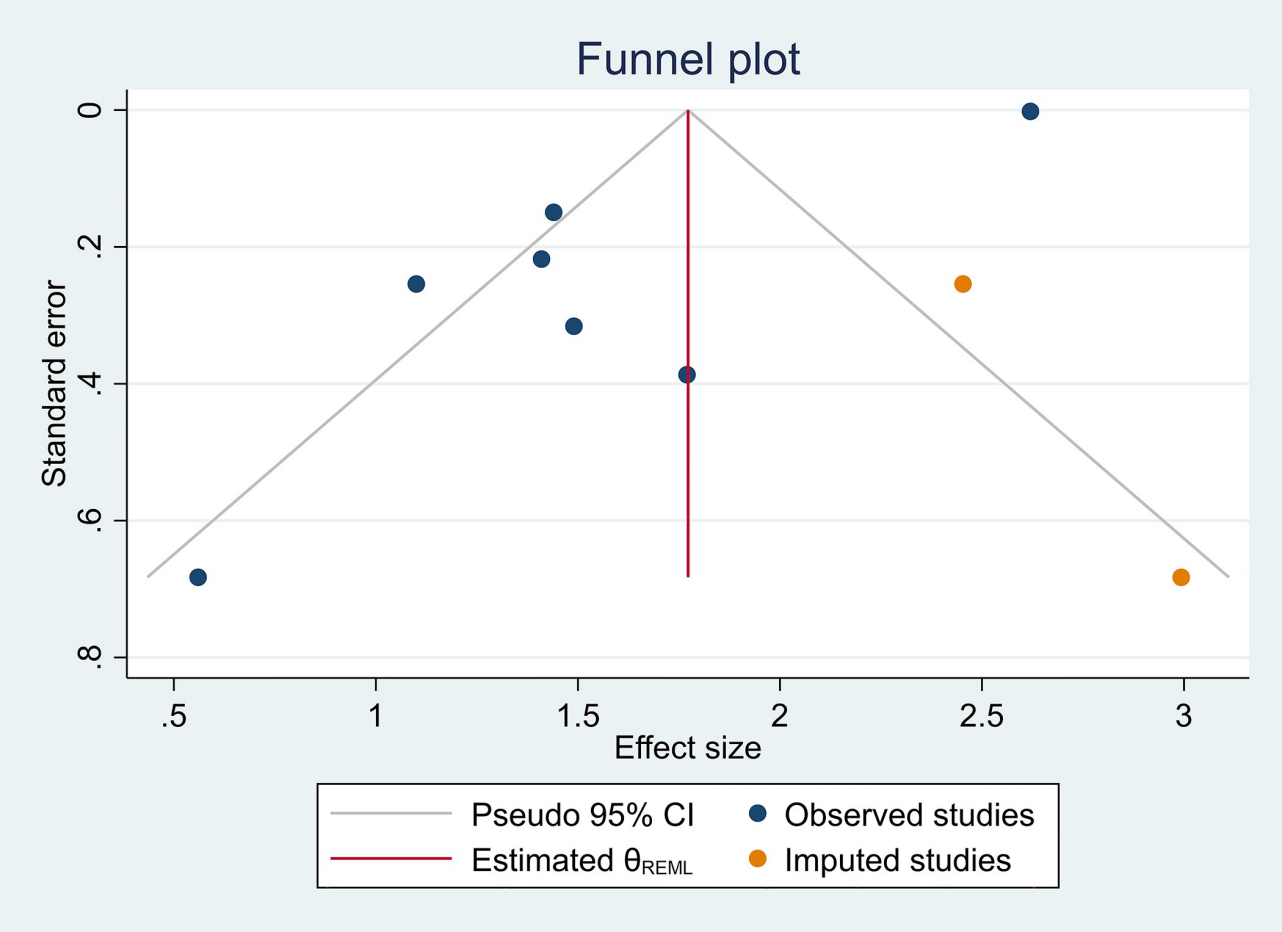
Funnel plot, observed and imputed studies.

We delve into the examination of heterogeneity and sensitivity analysis, employing the Galbraith plot as a visual aid to illuminate our findings (Fig 3). The Galbraith plot, a valuable tool in meta-analysis, assists in identifying potential outliers and evaluate the impact of individual studies on the overall pooled estimate. Our investigation revealed an I2 value of 91.34%, indicating a substantial degree of heterogeneity across the included studies. This high level of heterogeneity suggests that the variability among the study outcomes exceeds what would be expected by chance alone. Further, the H2 statistic, calculated at 11.55, reinforces the presence of heterogeneity, emphasizing the need for a careful exploration of potential sources contributing to the observed variability. To rigorously evaluate the differences between groups, a Test of group differences was conducted, yielding a Q_b statistic of chi2(5) = 155.02. The associated probability, Prob > Q_b = 0.000, underscores the statistical significance of group differences. This suggests that the observed heterogeneity is not merely a random occurrence but is indicative of systematic variations among the studies included in the meta-analysis. The Galbraith graph, coupled with these quantitative metrics, provides a comprehensive overview of the heterogeneity within our dataset. Researchers and readers alike can use this information to critically assess the robustness of our findings, guiding future investigations and potentially identifying influential studies that warrant closer scrutiny. As we navigate through the intricacies of heterogeneity and sensitivity analysis, the Galbraith graph emerges as a valuable instrument in unraveling the complexities inherent in our meta-analytical exploration. In addition to the Galbraith graph, we complemented our heterogeneity and sensitivity analysis by incorporating a funnel plot, a widely employed visualization tool in meta-analysis. The funnel plot provides insights into potential publication bias and allows for the examination of imputed studies (Fig 4). By plotting the effect size against its standard error, the funnel plot can reveal asymmetry, indicating the presence of bias or other factors influencing study outcomes. The inclusion of imputed studies further enriches our analysis, accounting for any missing data and enhancing the robustness of our findings. The combination of the Galbraith plot and the funnel plot with imputed studies offers a more nuanced perspective on the heterogeneity within our dataset, empowering researchers and readers to critically assess the reliability and generalizability of our meta-analytical results.

### Publication bias

In the critical assessment of our meta-analysis, it is imperative to address the potential presence of publication bias, a phenomenon where the likelihood of a study being published is influenced by the nature and direction of its results. To scrutinize this aspect, we employed both the regression-based Egger test and Begg’s test, complemented by a visual inspection of the funnel plot (Fig 4). The regression-based Egger test, conducted under a random-effects model using the Restricted Maximum Likelihood (REML) method, assessed the presence of small-study effects. The null hypothesis (H0: beta1 = 0) posits no small-study effects. The observed beta1 value of -2.69, with a standard error (SE) of 1.091, yielded a z-statistic of -2.46, resulting in a probability (Prob > |z|) of 0.0137. This statistically significant result suggests the presence of small-study effects, indicating potential asymmetry in the distribution of effect sizes, which may be attributed to publication bias.

Furthermore, Begg’s test, examining small-study effects through Kendall’s score, revealed a score of 5.00, with a standard error of 6.658. The associated z-statistic of 0.60 and a probability (Prob > |z|) of 0.5480 suggest no significant evidence of small-study effects according to Begg’s test.

To visually supplement our analysis, we employed a funnel plot, a graphical representation of study precision against effect size. A symmetrical and evenly distributed funnel plot generally indicates a lack of publication bias, while asymmetry may signal its presence.

In conclusion, while Begg’s test did not find compelling evidence of publication bias, the regression-based Egger test hinted at the possibility of small-study effects. The observed asymmetry in the funnel plot provides further context to these statistical tests, emphasizing the need for cautious interpretation of our findings. Researchers and readers are encouraged to consider the potential impact of publication bias on our results and exercise prudence when drawing conclusions from this meta-analysis.

## Discussion

Our systematic review and meta-analysis, focused on elucidating the potential association between wood dust exposure and the risk of leukemia, bring forth significant findings that warrant thorough consideration. The calculated overall odds ratio (OR) of 1.56, with a 95% confidence interval (CI) of 1.15–2.12, paints a compelling picture of a positive relationship. As we delve into the nuances of these results, it becomes essential to scrutinize the intricacies of our study design, the inherent strengths and limitations, and the broader implications for occupational health and public policy.

Firstly, our meta-analysis is noteworthy for its comprehensive approach to synthesizing evidence from case-control studies exclusively. The decision to focus on this study design allowed us to capture detailed exposure histories, essential in understanding potential risk factors. This choice is particularly relevant in the context of wood dust exposure, where nuances such as specific wood types, processing methods, and cumulative exposure over time can significantly impact the observed association.

The overall OR of 1.56 suggests a significant positive association, indicating that individuals with a history of wood dust exposure may face an increased risk of developing leukemia. This finding holds weight, especially considering the diverse range of wood-related industries and environmental settings where such exposures occur. Our study, therefore, contributes to the growing body of evidence that informs occupational health practices and public policy.

Addressing the potential influence of publication bias is paramount in any meta-analysis. While we employed a comprehensive search strategy, included unpublished studies, and considered gray literature, the possibility of unreported or unpublished studies with null findings impacting our overall conclusion cannot be entirely ruled out. Sensitivity analyses represent a valuable tool to assess the robustness of our findings. By exploring the impact of varying inclusion criteria or excluding specific studies, sensitivity analyses allow us to gauge the stability of the observed association. Future meta-analyses on this topic should consider incorporating these analyses to provide a more comprehensive evaluation of the potential impact of publication bias.

The identification of a positive association between wood dust exposure and the risk of leukemia has immediate and tangible implications for occupational health practices. Occupations such as woodworking, furniture manufacturing, and other wood-related industries are prevalent globally, and individuals working in these sectors face potential exposure to wood dust on a regular basis [20–24]. Occupational health practitioners should be attentive to the potential risks associated with wood dust exposure and incorporate these findings into health surveillance programs. Regular health monitoring, including comprehensive medical examinations and biomarker assessments, could aid in early detection and intervention [25]. The nature of wood dust exposure necessitates a proactive approach, with employers in wood-related industries prioritizing the implementation and enforcement of stringent safety protocols. Promoting the use of personal protective equipment, improving ventilation systems to minimize airborne wood dust particles, and establishing designated clean areas for breaks are practical measures that can significantly reduce the risk of exposure. Educational programs and awareness campaigns can further inform workers and employers about the potential health risks associated with wood dust, fostering a culture of prevention in these industries [26].

While our study design allowed us to capture detailed exposure histories, it is essential to acknowledge the inherent limitations of case-control studies. The retrospective nature of these investigations introduces the possibility of recall bias, where participants with leukemia may recall or report exposures differently compared to the control group. In the context of wood dust exposure, this bias could manifest if cases are more likely to remember or attribute their illness to occupational exposures than controls. Furthermore, the challenges of establishing temporality and causality in case-control designs necessitate cautious interpretation of our findings. Establishing a causal relationship between wood dust exposure and leukemia requires careful consideration of exposure timing concerning disease onset. Future case-control studies could benefit from incorporating more detailed exposure assessment techniques, such as cumulative exposure measures and exploration of potential latency periods. The reliance on self-reported exposure data may compromise the accuracy of exposure assessments. Efforts were made to account for this bias in the study design and analyses, but it remains an inherent limitation of case-control studies. Additionally, variations in case definitions, control selection, and exposure assessment methods among the included studies may contribute to the observed heterogeneity.

### Future research directions

Moving forward, our study provides a solid foundation for future research endeavors that aim to refine and expand our understanding of the association between wood dust exposure and leukemia. The following directions could guide researchers in further unraveling the complexities of this relationship:

1. Refinement of Exposure Assessment: Future studies should focus on refining exposure assessments within the context of case-control designs. Incorporating more detailed exposure histories, exploring job-exposure matrices, and integrating biomarkers of exposure could enhance the precision of exposure measurement [27].

2. Exploration of Temporal Aspects: Considering the temporal aspects of wood dust exposure is crucial. Future studies, especially case-control designs, should incorporate more detailed temporal aspects of exposure, considering cumulative exposure over time and potential latency periods [28].

3. Integration of Molecular Epidemiology: Molecular epidemiology approaches could provide insights into the genetic factors that may modulate the association between wood dust exposure and leukemia risk. Integrating genetic and molecular perspectives within case-control designs could enhance our understanding of individual susceptibility and mechanistic pathways [29].

4. International Collaboration: Collaborative efforts across research institutions globally are essential. Pooling data from diverse populations and settings could enhance the generalizability of findings and provide a more comprehensive understanding of the association.

5. Impact of Intervention Strategies: Assessing the effectiveness of intervention strategies aimed at reducing wood dust exposure is vital. Long-term follow-up studies, including case-control designs, could compare leukemia incidence in populations before and after the implementation of preventive measures.

6. Comprehensive Meta-analyses: Future meta-analyses should continue to refine our understanding of the association. Subgroup analyses should be expanded to explore additional sources of heterogeneity, and meta-regression analyses can further quantify the impact of specific study characteristics on effect sizes.

## Conclusion

In conclusion, our systematic review and meta-analysis investigating the association between wood dust exposure and the risk of leukemia present compelling evidence that warrants attention from both the scientific community and stakeholders in occupational health and safety. The calculated overall odds ratio (OR) of 1.56, with a 95% confidence interval (CI) of 1.15–2.12, signifies a statistically significant positive association, suggesting an increased risk of leukemia among individuals with a history of wood dust exposure. The robustness of our findings is underpinned by a comprehensive assessment of case-control studies, utilizing The Joanna Briggs Institute Critical Appraisal tools, all of which were determined to be at low risk of bias. This meticulous quality assessment enhances the reliability of the evidence synthesized in our meta-analysis, contributing to the overall strength of our study. The observed heterogeneity in our meta-analysis, as indicated by an I^2 of 87%, underscores the diverse nature of the included studies. Subgroup analyses shed light on potential sources of variability, such as study design, geographic location, and the type of wood dust exposure. However, the complexity of wood dust, with variations in exposure assessment methodologies and specific characteristics across different wood types and processing methods, remains a challenge. Future research should delve deeper into these nuances to unravel the intricacies of the association. Our study has immediate implications for occupational health practices and public policy. The positive association identified emphasizes the need for proactive measures in workplaces where wood dust exposure is prevalent. Occupational health practitioners should integrate these findings into health surveillance programs, incorporating regular health monitoring and biomarker assessments. Employers in wood-related industries must prioritize the implementation of stringent safety protocols, including the use of personal protective equipment and advanced ventilation systems. The study’s strengths lie not only in the meticulous methodology employed but also in the integration of a comprehensive quality assessment process. The use of standardized tools ensures a systematic evaluation of each included study, providing transparency in the strengths and limitations of the evidence synthesized. Sensitivity analyses and careful consideration of potential sources of bias contribute to the robustness of our conclusions. While our study significantly advances our understanding of the association between wood dust exposure and leukemia, it is essential to acknowledge its limitations. The retrospective nature of many included studies introduces the possibility of recall bias, and variations in diagnostic criteria for leukemia across studies may contribute to heterogeneity. The generalizability of our findings should be considered in the context of the specific populations and methodologies included in this meta-analysis. In light of these considerations, our study calls for continued research efforts to refine our understanding of the association. Future investigations should explore specific wood types and processing methods, incorporate more refined exposure assessments, and consider the temporal aspects of exposure. Collaboration across international research institutions, sharing data and resources, will be instrumental in conducting larger-scale analyses that enhance the generalizability of findings. In the broader context of occupational health and public policy, our findings underscore the importance of evidence-based interventions and regulations to safeguard the well-being of individuals exposed to wood dust. The integration of preventive measures in workplaces, informed by the insights gained from this study, is crucial for minimizing the potential health risks associated with wood dust exposure.

In conclusion, our systematic review and meta-analysis contribute to the growing body of evidence supporting a positive association between wood dust exposure and the risk of leukemia. While providing valuable insights, this study should be viewed as part of an ongoing scientific discourse. As we collectively strive for a deeper understanding of occupational hazards, our findings serve as a stepping stone toward informed policies, improved preventive strategies, and ultimately, enhanced health outcomes for individuals working in wood-related industries worldwide.

## Supporting information

S1 File PRISMA checklist. (DOC)

S2 File Included articles. (RAR)
